# Prognostic Utility of HEFESTOS Score and Complementary Lung Ultrasound for Heart Failure Decompensation in Primary Care Outpatients: A Prospective Cohort Study

**DOI:** 10.3390/jcdd12090347

**Published:** 2025-09-11

**Authors:** Marcos Haro-Montoya, Rosa Caballol-Angelats, José Fernández-Sáez, Maylin Montelongo-Sol, Laura Conangla-Ferrin, Victoria Cendrós-Cámara, Jose María Verdú-Rotellar, Josep Lluís Clua-Espuny

**Affiliations:** 1 Unitat Docent de Medicina Familiar i Comunitària, Gerència d’Atenció Primària i a la Comunitat, Tortosa-Terres de l’Ebre, Institut Català de la Salut, 43500 Tortosa, Spain; mharom@sescam.jccm.es; 2 Zona Básica de Salud de Iniesta, Consultorio de Ledaña, Gerencia de Atención Integrada de Albacete, Servicio de Salud de Castilla-La Mancha, 16237 Ledaña, Spain; 3 Equip d’Atenció Primària Tortosa Est, Gerència d’Atenció Primària i a la Comunitat de les Terres de l’Ebre, Institut Català de la Salut, 43500 Tortosa, Spain; mmontelongo.ebre.ics@gencat.cat; 4 Grup de Recerca ecoAP, Fundació Institut Universitari per a la Recerca a l’Atenció Primària de Salut Jordi Gol i Gurina (IDIAPJGol), 08007 Barcelona, Spain; lconanglamn.ics@gencat.cat; 5 Unitat de Suport a la Recerca de Terres de l’Ebre, Fundació Institut Universitari per a la Recerca a l’Atenció Primària de Salut Jordi Gol i Gurina (IDIAPJGol), 43500 Tortosa, Spain; jfernandezsa.ebre.ics@gencat.cat; 6 Equip d’Atenció Primària Badalona Centre i Dalt la Vila, Gerència d’Atenció Primària i a la Comunitat Barcelonès Nord i Maresme, Institut Català de la Salut, 08911 Badalona, Spain; 7 Departament de Medicina, Universitat de Barcelona, 08036 Barcelona, Spain; vcendros.bcn.ics@gencat.cat; 8 Unitat de Suport a la Recerca de Barcelona, Fundació Institut Universitari per a la Recerca a l’Atenció Primària de Salut Jordi Gol i Gurina (IDIAPJGol), 08007 Barcelona, Spain; verdujm@gmail.com; 9 Equip d’Atenció Primària Adrià, Gerència d’Atenció Primària i a la Comunitat Barcelona Litoral Esquerre, Institut Català de la Salut, 08021 Barcelona, Spain; 10 Equip d’Atenció Primària Sant Marti, Gerència d’Atenció Primària i a la Comunitat Barcelona Litoral Esquerre, Institut Català de la Salut, 08020 Barcelona, Spain

**Keywords:** heart failure, decompensation, primary care, ultrasonography, risk stratification

## Abstract

Heart failure (HF) is a major contributor to morbidity, mortality, and healthcare costs, particularly among older adults. Effective outpatient risk stratification remains a clinical challenge, especially following hospital discharge or episodes of acute decompensation. Although both lung ultrasound (LUS) and the HEFESTOS score have shown individual prognostic value, their combined use in primary care settings has not been extensively explored. This prospective cohort study included 107 patients with confirmed HF followed at a primary care center in southern Catalonia. At baseline, all patients underwent LUS and HEFESTOS assessment. The primary outcome was HF decompensation, defined as worsening symptoms requiring medical attention, emergency care, hospitalization, or death. Over a mean follow-up of 72 days, 25 patients (23.3%) experienced decompensation. In multivariate analysis, only the HEFESTOS score was independently associated with decompensation. LUS and HEFESTOS showed moderate agreement (Kappa = 0.456), and LUS demonstrated moderate discriminative capacity (AUC = 0.677) with high sensitivity (81.7%) and positive predictive value (81.7%). These findings support the routine use of the HEFESTOS score in primary care and suggest that LUS may serve as a complementary tool, particularly for identifying subclinical pulmonary congestion. Their combined use could enhance outpatient risk stratification and guide individualized follow-up strategies in HF management.

## 1. Introduction

Heart failure (HF) represents one of the chronic conditions with the greatest global health and economic impact. It is estimated to affect approximately 60 to 64.3 million people worldwide [1,2]. This prevalence exhibits marked variability in epidemiological studies, significantly increasing with age and exceeding 10% in individuals over 70 years old and 14% in those over 75 years old [3,4,5,6].

Beyond its clinical consequences, HF imposes a considerable economic burden on healthcare systems. HF remains the leading cause of hospitalization in patients aged 65 years or older. Furthermore, it is recognized as the third leading cause of cardiovascular mortality, contributing up to 20% of deaths from this cause. Compounding this situation, annual rehospitalization rates for HF patients can reach up to 50%. Each new hospitalization not only severely deteriorates patients’ quality of life but also independently increases the risk of mortality by 20%. This hospitalization–mortality cycle represents a critical vulnerability in the current HF care pathway. The high rate of rehospitalizations, often triggered by acute HF decompensation, is a key factor contributing to the economic burden and morbidity of the disease. Recent data from Spain confirm this burden, with in-hospital mortality and readmission rates remaining alarmingly high despite advances in pharmacological treatment [5].

Despite this reality, a significant proportion, nearly 40%, of patients are discharged prematurely after an HF hospitalization [7]. This situation highlights a critical discontinuity in the continuum of care, suggesting that existing discharge criteria, patient education, or post-discharge support mechanisms are often insufficient to prevent early adverse events, including symptomatic worsening or rehospitalizations. The current system’s inability to effectively manage patients after discharge results in a considerable financial burden on healthcare systems, as recurrent hospitalizations are costly. More importantly, it imposes a profound human cost, leading to repeated deterioration in patients’ quality of life and increased mortality. This precarious situation creates an urgent need to implement additional and more effective measures for the outpatient follow-up of HF patients. Robust outpatient monitoring is crucial to bridge the gap between acute hospital care and stable long-term management, with the ultimate goal of reducing rehospitalizations, improving patient quality of life, and lowering overall healthcare costs.

In this context, lung ultrasound (LUS) has rapidly emerged as a non-invasive, fast, and accurate tool for recognizing and monitoring pulmonary congestion in patients with heart failure for detecting pulmonary congestion, primarily through the identification of B-lines [8,9]. The utility of LUS extends beyond initial diagnosis; it is increasingly used to guide diuretic therapy, monitor congestion resolution during treatment, and predict adverse outcomes. Its diagnostic and prognostic value in the outpatient setting has been supported by studies showing strong correlation with natriuretic peptides and echocardiographic parameters [10,11]. Its bedside applicability is well established in various settings, including emergency departments, critical care units, and outpatient clinics [12,13]. LUS offers a unique opportunity to shift the paradigm of HF management from reactive (treating overt decompensation) to proactive (preventing decompensation). By enabling the detection of subclinical pulmonary congestion, LUS is a substantial improvement over subjective clinical assessment [14]. However, international clinical practice guidelines on heart failure still do not incorporate standardized interpretation criteria for the predictive value of B-lines [15,16]. Challenges persist for its widespread adoption and consistent application, including operator dependency, variability in B-line interpretation, equipment differences, and the inherent difficulty in distinguishing overlapping pulmonary conditions. Consequently, physician adoption and interpretation of LUS findings remain suboptimal.

In parallel with advances in imaging modalities, the HEFESTOS score has been validated in several European countries as a useful predictive model in primary care for stratifying the risk of death or hospitalization within 30 days post-cardiac decompensation [13]. A key strength of the HEFESTOS score lies in its reliance on clinical variables that are easily accessible and obtainable in a community setting, making it highly practical for routine use in primary care. The score’s primary utility lies in its ability to guide clinical decision-making by providing a quantitative, evidence-based assessment of short-term prognosis for patients experiencing HF decompensation in the primary care setting. This addresses a critical unmet need in primary care: the ability to quickly identify patients at high risk of adverse outcomes immediately following a decompensation event.

Currently, there is a significant gap in scientific evidence regarding the efficacy of B-lines (detected by LUS) as a hospital discharge criterion in patients admitted for decompensated HF or in outpatient follow-up [12,13]. Similarly, the prognostic relevance of combining B-lines with other clinical scores, such as the HEFESTOS score, in monitoring HF patients is unknown. Furthermore, there is a notable absence of specific indications or guidelines for leveraging the predictive value of their combined use in guiding outpatient therapeutic interventions during HF decompensation episodes. This lack of evidence on combining LUS (an objective physiological measure of congestion) and HEFESTOS (a clinical risk score based on historical and clinical factors) suggests that an opportunity for a more comprehensive and nuanced risk assessment is being missed. The potential of combining multiparametric ultrasound strategies with clinical scores to improve risk stratification in HF has been recently highlighted in outpatient cohorts [17].

This study proposes to evaluate the efficacy of this combined use of LUS and the HEFESTOS score during primary care follow-up will reduce the combined risk of symptomatic worsening and/or adverse events in heart failure patients.

This study aimed to analyze the characteristics and clinical course of patients diagnosed with HF through the combined use of the HEFESTOS score and LUS. Specifically, we sought to (1) evaluate the prognostic utility of the HEFESTOS score beyond the 30-day follow-up period; (2) analyze the prognostic significance of LUS findings during HF follow-up; and (3) determine the concordance between the degree of pulmonary congestion detected by LUS and the HEFESTOS-based risk stratification for hospitalization or death.

## 2. Materials and Methods

This study is designed as an observational, prospective cohort study with a longitudinal and analytical approach. It involves the systematic follow-up of a group of patients over time, allowing for the recording and analysis of clinical outcomes and their association with baseline characteristics and diagnostic tools applied at the outset. The main objective was to evaluate the combined use of the HEFESTOS score and lung ultrasound for managing HF patients in an outpatient setting. The primary outcome measured was HF decompensation, which was defined as worsening symptoms requiring medical attention, emergency care, hospitalization, or death.

At inclusion, each patient underwent a baseline assessment that included the application of the HEFESTOS risk calculator and a LUS (LUS) examination. Follow-up was carried out through regular review of electronic health records (EHRs) to identify episodes of heart failure decompensation, including primary care consultations, emergency department visits, hospital admissions, and deaths.

### 2.1. Setting

The study was conducted at the “EAP Tortosa-Est—CAP El Temple” primary care center, part of the public healthcare network of the Spanish National Health System, managed by the Institut Català de la Salut (ICS) within the Catalan Health System (CatSalut). This center is located in the city of Tortosa and provides healthcare to a predominantly urban population. An EAP (Equip d’Atenció Primària) is a functional unit of the Catalan public health system, composed of a multidisciplinary team of healthcare professionals.

Tortosa is situated in the southernmost region of Catalonia, known as *Terres de l’Ebre. Terres de l’Ebre* is characterized by a markedly aging population, with the highest aging index in Catalonia (174.0), compared to the Catalan average (142.6) [18] and the Spanish average (142.3) [19]. Furthermore, the average income per inhabitant in the region represents only 78.1% of the Catalan average, reflecting notable socioeconomic disparities [20].

### 2.2. Inclusion Criteria

Age ≥ 18 years. Participants had to be adults, defined as individuals aged 18 years or older at the time of inclusion in the study, to ensure legal capacity for providing informed consent and to reflect the typical age range of heart failure patients managed in primary care.

Documented diagnosis of heart failure. Only patients with a confirmed diagnosis of heart failure recorded in their electronic health records were eligible. This included both heart failure with reduced ejection fraction (HFrEF) and heart failure with preserved ejection fraction (HFpEF), as coded using standard ICD-10 classifications. All patients were required to have undergone a transthoracic echocardiogram to support diagnosis and classification, following the diagnostic criteria established by the European Society of Cardiology (ESC) guidelines [9].

Ability to provide informed consent. Participants had to demonstrate sufficient cognitive and communicative capacity to understand the nature and objectives of the study, and to voluntarily agree to participate by signing an informed consent form.

Patients with a documented diagnosis of heart failure, coded using standard ICD-10 classifications, were identified through a review of electronic health records. Those who met the inclusion criteria were subsequently contacted by telephone and invited to participate in the study on a voluntary basis.

### 2.3. Exclusion Criteria

Patients enrolled in the ATDOM (home care) program. These individuals typically present with significant functional dependence, complex chronic conditions, or severe mobility limitations that prevent regular attendance at the primary care center. Although portable ultrasound devices were available, these patients were excluded due to the clinical and logistical complexity of ensuring consistent follow-up.

Patients identified as MACA (*Malaltia Crònica Avançada*) according to the criteria established by the Catalan Health Department [21]. These patients present advanced chronic conditions with limited life expectancy, high clinical complexity, and are primarily candidates for palliative care rather than active monitoring of decompensation risk.

Refusal to participate or inability to provide informed consent (e.g., cognitive impairment without a legal representative).

### 2.4. Study Period

The inclusion of patients in the cohort was carried out progressively between April 2023 and April 2025, alongside routine clinical practice at the EAP Tortosa-Est primary care center. Each patient underwent an initial assessment with LUS and the HEFESTOS risk calculator at the time of inclusion. In 2025, electronic health records were reviewed retrospectively to identify the first episode of HF decompensation occurring after each patient’s inclusion in the study. Patients without any recorded decompensation were followed administratively up to April 2025.

### 2.5. Informed Consent

All participants provided written informed consent prior to their inclusion in the study. The study protocol was reviewed and approved by the Clinical Research Ethics Committee of IDIAP Jordi Gol (reference 22/143P). Participants were informed about the objectives, procedures, potential risks, and benefits of the study, and their right to withdraw at any time without affecting the quality of their medical care.

### 2.6. Variables Collected

Several clinical and diagnostic variables were recorded for each patient at baseline and during follow-up, with the aim of characterizing the cohort and identifying potential predictors of HF decompensation.

Sociodemographic and clinical characteristics: Age and sex were recorded, as well as the etiology of HF (underlying cause, such as ischemic heart disease or hypertension) and the type of heart failure (HFrEF or HFpEF), as documented in the electronic health record. The baseline evaluation for the study, including LUS and HEFESTOS score calculation was performed on the date of each patient’s inclusion.

Lung Ultrasound (LUS) Score: The main ultrasonographic marker of pulmonary congestion is the B-line, a vertical hyperechoic artifact that originates at the pleural line and extends down to the bottom of the screen without fading, moving synchronously with respiration. These artifacts are generated by the reverberation of ultrasound waves at air-fluid interfaces in the lung, typically in the setting of interstitial edema. In this study, the chest was examined following a standardized 28-zone protocol [22], scanning anterior, lateral, and posterior regions of both lungs. The total number of B-lines observed across all zones was used to quantify pulmonary congestion and assign a LUS score, which was classified into three categories: mild (0–14 B-lines), moderate (15–29 B-lines), and severe congestion (≥30 B-lines). A detailed description of the LUS scanning protocol, including patient positioning, probe type, scanning technique, and B-line grading criteria, is provided in Table 1.

HEFESTOS Risk Score: HEFESTOS score is a validated clinical tool used to estimate the short-term (30-day) risk of HF decompensation [18]. It is based on 8 variables related to the patient’s symptoms, vital signs, and treatment at the time of consultation: gender; history of hospital admission due to HF within the past year (yes or no); heart rate (≤100 or >100 beats per minute); presence of pulmonary crackles on auscultation (yes or no); paroxysmal nocturnal dyspnea (yes or no); orthopnea (yes or no); New York Heart Association (NYHA) functional class (I or II vs. III or IV); dyspnea as the main reason for the medical encounter (yes or no); and oxygen saturation measured by pulse oximetry (≤90% or >90%). Each item scores 1 point, with a maximum total of 8 points. Based on the total score, patients are stratified into three risk categories—low risk (0–3 points), with an estimated 30-day risk of decompensation <5%; moderate risk (4–7 points), with an estimated risk of 5–20%; and high risk (>7 points), with an estimated risk >20%—as shown in Table 2.

Heart failure decompensation: HF decompensation is defined as a clinical worsening of a previously stable patient, usually due to fluid overload, increased pulmonary or systemic congestion, or inadequate cardiac output. These episodes typically require a change in medical management or healthcare utilization. In this study, decompensation was identified through retrospective review of electronic health records and included any of the following events occurring after the date of inclusion:

- Consultations in primary care for symptoms consistent with HF worsening (e.g., dyspnea, orthopnea, weight gain, peripheral edema).
- Emergency department visits where HF was noted as the main reason for consultation or part of the diagnostic impression.
- Hospital admissions with a primary or secondary diagnosis of HF decompensation, coded according to standard ICD-10 classifications.
- All-cause mortality, whether related or unrelated to HF, as recorded in the health system database.

Only the first decompensation event per patient after their inclusion was considered for analysis, in line with the study’s objective of evaluating predictive tools at baseline.

## 3. Results

A total of 107 patients with HF (HF) were included. The mean age was 77.3 years, and 55.1% were male. HF with preserved ejection fraction (HFpEF) was more frequent (55.1%) than with reduced ejection fraction (HFrEF) (45.8%). The main etiologies were hypertension (62.06%), arrhythmias (44.94%), and ischemic heart disease (24.61%). A detailed overview of the participants’ demographic and clinical characteristics is presented in Table 3.

**Figure 1:**
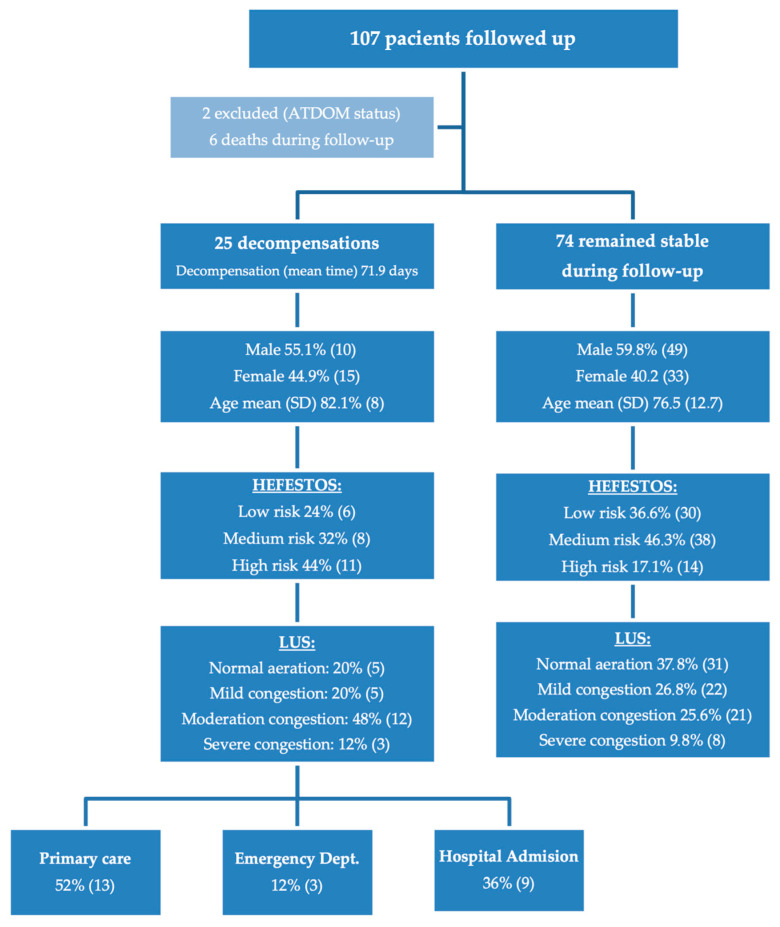
Flow-chart during follow-up.

During the follow-up period, 25 patients (23.36%) experienced cardiac decompensation (Figure 1). This included 16.9% of men (95% CI: 6.52–27.27%) and 31.2% of women (95% CI: 17.09–45.4%). No statistically significant differences were observed between sexes (*p* = 0.131).

In the initial evaluation, according to the HEFESTOS score, 36 patients (33.6%) were classified as low risk, 46 (43.0%) as medium risk, and 25 (23.4%) as high risk. Regarding the initial LUS assessment, 36 patients (33.6%) showed an aerated lung pattern, 27 (25.2%) presented a mild interstitial pattern, 33 (30.8%) had a moderate interstitial pattern, and 11 patients (10.3%) displayed a severe interstitial pattern. A summary of the risk stratification based on HEFESTOS and the initial LUS findings is provided in Table 4.

To evaluate the agreement between HEFESTOS risk classification and LUS findings, both tools were dichotomized clinically: HEFESTOS into “Low risk” and “Medium–high risk”, and LUS into “Normal Aereation and Mild congestión” and “Moderate and severe congestion” (Table 5). The unweighted Cohen’s Kappa index yielded a value of 0.456 (95% CI: 0.315–0.597; *p* < 0.001), indicating a moderate level of agreement between the two tools. The predictive power of using both scores is statistically significant (*p* = 0.005), with their primary utility being in the stratification of low-risk individuals.

The area under the curve (AUC) was 0.677 (95% CI: 0.552–0.801; *p* < 0.005), demonstrating moderate discriminative capacity. Sensitivity was 81.7% (95% CI: 72.7–90.7), specificity 63.9% (95% CI: 48.2–79.6), positive predictive value 81.7%, and negative predictive value 63.9%, with the same confidence intervals. In our sample, the positive predictive value (PPV) and negative predictive value (NPV) were numerically identical to sensitivity and specificity, respectively (PPV = 81.7%, NPV = 63.9%), due to the symmetry of the contingency table (FP = FN). We emphasize that these are empirical estimates, specific to the cohort prevalence (66.4%), and not generalizable to populations with different prevalence.

The correlation between the total LUS score and the HEFESTOS scale was also assessed, showing a Pearson correlation coefficient of 0.489, a Spearman rank correlation of 0.514, and an intraclass correlation coefficient (ICC) of 0.390—suggesting a low to moderate correlation between the two continuous measures.

In the group of 25 patients with decompensated HF, a significant association was observed between LUS findings and the HEFESTOS risk score. Among the 10 patients with an aerated or mild pattern on LUS, 6 were classified as low risk by HEFESTOS and 4 as moderate or high risk. Conversely, all 15 patients with a moderate or severe LUS pattern were categorized as moderate or high risk according to HEFESTOS. This corresponded to a sensitivity of 78.95% (95% CI: 60.62–97.28) and a specificity of 100% (95% CI: 86.07–100.00) for LUS in detecting patients at moderate-to-high risk based on the HEFESTOS score. The positive predictive value (PPV) was 100%, and the negative predictive value (NPV) was 60.0% (95% CI: 29.64–90.36).

In the multivariate model with decompensation as the dependent variable, which included age, sex, HEFESTOS risk, and LUS score, only the HEFESTOS risk maintained a statistically significant association with decompensation (95% CI [1.01–1.06]; *p* = 0.01). LUS score, age, and sex were not statistically significant.

Table 6 summarizes the statistical analyses comparing the HEFESTOS risk classification with LUS findings, including measures of agreement, diagnostic accuracy, correlation, and multivariate predictive value.

## 4. Discussion

In our primary care cohort, the main etiologies were hypertension (62.1%), tachyarrhythmias (44.9%, a category that included all patients with atrial fibrillation, even if tachyarrhythmia was not present at the time of evaluation), and ischemic cardiomyopathy (24.6%). Atrial fibrillation is well recognized as the second most common precipitant of HF decompensation after acute coronary syndromes, which helps explain the relatively high proportion observed in our sample. This distribution reflects the characteristics of our study population and does not necessarily represent the overall epidemiology of HF.

Findings included that 25 patients (23.3%) experienced decompensation during a mean follow-up of 72 days. The HEFESTOS score was found to be a statistically significant predictor of decompensation in a multivariate analysis, supporting its prognostic value beyond the initial 30-day period. LUS demonstrated moderate discriminative capacity (AUC = 0.677), with high sensitivity (81.7%) and positive predictive value (81.7%) in identifying patients at higher risk. The study also found a moderate agreement (Cohen’s Kappa = 0.456) and a low to moderate correlation (Pearson coefficient = 0.489) between the HEFESTOS and LUS scores.

Natriuretic peptides remain the gold-standard biomarker in HF diagnosis and prognosis. However, in primary care, access to NT-proBNP testing may be limited. In this context, tools such as LUS and the HEFESTOS score, provide rapid and pragmatic alternatives. Importantly, these tools should not be seen as substitutes but as complementary to NT-proBNP in a multimodal approach to risk stratification. LUS is a rapid, mobile, and non-invasive method to monitor pulmonary congestion and identify high-risk patients [23,24,25,26], with greater accuracy than physical examination or chest X-ray [27,28], and it adds prognostic value when combined with neuropeptides and novel biomarkers such as CA19 [9,29,30,31]. However, despite these benefits, LUS is not yet systematically implemented in primary care. Current ESC Guidelines (Class I, level B) recommend intensive early treatment and close follow-up after HF hospitalization [9,32], but they do not specifically endorse LUS for detecting pulmonary congestion in outpatients.

The updated consensus underscores the recent advances in LUS and provides a contemporary framework for its clinical use. The consensus statement of the European Association of Cardiovascular Imaging [33] is now considered the reference standard in HF care, yet additional research is still required, particularly for outpatient monitoring in primary care. Although the literature does not explicitly address the combined use of the HEFESTOS score and LUS, both tools target similar clinical domains and patient profiles, especially in the context of HF decompensation. The HEFESTOS score offers a simple and validated method for identifying high-risk patients based on clinical history and symptoms, making it highly applicable in primary care, whereas LUS provides an immediate, objective, and dynamic assessment of pulmonary congestion that often precedes overt symptoms. Our findings suggest a synergistic value when both tools are applied together, allowing for a pragmatic decision-making framework. When both HEFESTOS and LUS indicate low risk (HEFESTOS ≤ 3, aerated or mildly congested lung), patients may be safely managed with routine follow-up. If LUS shows moderate-to-severe congestion despite a low HEFESTOS score, clinicians should suspect subclinical congestion and consider early treatment intensification. Conversely, a high HEFESTOS score with minimal LUS findings may reflect risk driven by non-congestive mechanisms, warranting closer surveillance and short-term reassessment. Table 7 summarizes this proposed clinical algorithm and its follow-up recommendations according to the combined results of HEFESTOS and LUS at baseline. Importantly, this strategy should be interpreted as a complement—not a substitute—to the initiation or titration of guideline-directed medical therapy (GDMT), fully aligned with ESC guideline recommendations, while providing a practical framework to tailor follow-up intensity based on short-term risk stratification in the outpatient setting.

Moreover, because LUS can be repeated easily and interpreted dynamically, its use is particularly valuable in transitional care programs or in patients flagged as high risk by HEFESTOS. This dual-strategy offers a nuanced and individualized approach to risk assessment, bridging clinical scoring with physiologic monitoring to better anticipate—and potentially prevent—heart failure decompensation.

By designing the study to be inclusive of all HF phenotypes, regardless of ejection fraction, the research seeks to establish a universally applicable management strategy. This is particularly crucial for HFpEF patients, for whom effective, evidence-based therapies are still less defined compared to HFrEF. Demonstrating efficacy across the spectrum of ventricular function would significantly enhance the generalizability and clinical relevance of the study’s findings, making the proposed intervention a more robust and widely adoptable approach for the diverse and growing HF patient population.

While NT-proBNP and LUS are commonly used to diagnose and manage heart failure, several studies have explored their incremental value [10,34,35,36], particularly in emergency settings. Combining both tools provides a more comprehensive assessment than using either alone: NT-proBNP reflects cardiac stress, whereas LUS offers real-time imaging of pulmonary congestion. Evidence suggests that the prognostic value of LUS B-lines can be comparable to NT-proBNP, especially in HFpEF, highlighting their complementary role in diagnosis and follow-up. However, our study did not include NT-proBNP data, as it was specifically designed to evaluate the combined use of the HEFESTOS score and LUS in the outpatient setting. We acknowledge this as a limitation and recommend direct comparisons with NT-proBNP as an important future line of research.

The study has several limitations that affect the interpretation and generalizability of its findings. On one hand, the study population consisted of 107 patients. While this allowed for group comparisons, the small sample size may limit the statistical power and the ability to generalize the results to a broader population. On other hand, the study found a statistically significant positive correlation between both scores, but the correlation coefficients were moderate. This indicates that while a relationship exists, suggesting other factors are far more influential. Finally, the use of the LUS scale for B-line quantification, while a standard clinical tool, is a rating-based method that may be less sensitive or objective due to its dependence on the individual clinical experience of the operator.

## 5. Conclusions

The HEFESTOS score was independently associated with heart failure decompensation in this cohort, confirming its prognostic relevance beyond the initial 30-day timeframe and supporting its routine use for risk stratification in the outpatient setting.

Although lung ultrasound (LUS) did not retain independent prognostic value in multivariate analysis, it showed good sensitivity and moderate discriminative ability. Its capacity to detect pulmonary congestion and its moderate agreement with HEFESTOS suggest a valuable complementary role, particularly in cases of diagnostic uncertainty or for early identification of subclinical congestion.

These findings support the use of the HEFESTOS score in primary care and highlight the potential utility of integrating LUS into individualized follow-up strategies. Further studies are warranted to define the most effective ways to combine clinical scoring systems with physiologic monitoring tools in real-world HF management.

## Figures and Tables

**Table 1:** Lung Ultrasound B-line Assessment and Interpretation Protocol.

Parameter	Protocol Description
Probe	Convex probe (2–5 MHz) or Phased array (3.5–5 MHz)
Patient Position	Supine; seated position if clinically appropriate
Scanning Zones	28 intercostal spaces: anterior, lateral, and posterior regions bilaterally. Each hemithorax is divided into: Anterior region (anterior to the anterior axillary line) Lateral region (between the anterior and posterior axillary lines) Posterior region (posterior to the posterior axillary line)
Technique	Longitudinal scanning between intercostal spaces with the probe positioned perpendicular to the ribs. “Fanning” motion
B-line definition	Vertical and hyperechoic lines arising from pleura to the edge of the screen, moving synchronously with respiration
Congestion Grading	0–5 B-lines: Normal lung aeration 6–15: Mild congestion 16–30: Moderate >30 or confluent: Severe congestion

**Table 2:** HEFESTOS Score Components and Risk Stratification.

HEFESTOS Variables	Points (Additive Score)	Risk Groups and Estimated 30-Day Risk of Decompensation (%)	
Male	1	Low risk Score ≤3 points	<5%
Previous hospitalization	3		
Crackles	2		
Paroxysmal nocturnal dyspnoea	2		
Orthopnoea	2		
NYHA class III-IV	2		
Worsening in NYHA status	3		
Heart rate >100 b.p.m.	3		
Oxigen saturation <90%	5	Medium risk Scores = 4–7 points	5–20%

**Table 3:** Baseline Characteristics of Study Participants.

Variable	n (Mean)	% (SD)
Total	107	
Sex		
Male	59	55.14%
Female	48	44.86%
Age *(mean|SD)*	77.83 (mean)	12.02 (SD)
Mean time since HF diagnosis *(years)*	7.3	
HFpEF	59	55.14%
HFrEF	49	45.79%
Hypertensive heart disease	58	62.06%
Tachycardia-induced cardiomyopathy	42	44.94%
Ischemic cardiomyopathy	23	24.61%
Dilated cardiomyopathy	21	22.47%
Valvular heart disease	12	12.84%
Cardiac amyloidosis	3	3.21%

**Table 4:** Initial Risk Stratification According to HEFESTOS Score and Lung Ultrasound Findings.

Variable	n (Mean)	%(SD)
HEFESTOS risk (mean|SD)	5.26 (mean)	3.79 (SD)
Low risk (≤3 points)	36	33.64
Medium risk (4–7 points)	46	42.99
High risk (≥8 points)	25	26.36
LUS score (mean|SD)	14.32 (mean)	14.26 (SD)
Normal aereation (0–5 B-lines)	36	33.64
Mild congestion (6–15 B-lines)	27	25.23
Moderate congestion (16–30 B-lines)	33	30.84
Severe congestion (>30 B-lines)	11	10.28

**Table 5:** Crossed Risk Stratification According to HEFESTOS Score and Lung Ultrasound Findings (Moderate/Severe).

HEFESTOS Risk (Total Score)	LUS Score (B-Lines)				All	TOTAL
	N (<5)	Mild (6–15)	Mod (16–30)	Sev. (>30)		
Low (≤3) *n*_1_ = 36 (33.64%)	23	10	3	0	36	3/36; 8.3% (CI95% 1.75–22.4)
Medium (4–7) *n*_2_ *=* 46 (43%)	8	14	18	6	46	24/46; 52.1% (CI95% 36.6–67.7) *p* = 0.0001
High (≥ 8) *n*_3_= 25 (23.3%)	5	3	12	5	25	17/25; 68% (CI95% 47.7–88.2) *p* = 0.299
All	36	27	33	11	107	

**Table 6:** Summary of Agreement, Diagnostic Performance, and Prognostic Value of Lung Ultrasound Compared to the HEFESTOS Risk Classification.

Analysis	Indicator	Value
Agreement dichotomized classification	Cohen’s Kappa	0.456 (95% CI: 0.315–0.597); *p* < 0.001
Diagnostic performance	AUC	0.677 (95% CI: 0.552–0.801); *p* = 0.005
	Sensitivity	81.7% (95% CI: 72.7–90.7)
	Specificity	63.9% (95% CI: 48.2–79.6)
	PPV	81.7% (95% CI: 72.7–90.7)
	NPV	63.9% (95% CI: 48.2–79.6)
Correlation between continuous scores	Pearson correlation coef.	0.489
	Spearman rank correlation	0.514
	Intraclass correlation coef.	0.390
Multivariate model outcome: decompensation	HEFESTOS	OR 1.04 (95% IC: 1.01–1.06); *p* = 0.01
	LUS score	OR 1.01 (95% IC: 0.99–1.06); *p*= 0.152

**Table 7:** “Suggested Follow-Up Plan According to Combined HEFESTOS Score and Lung Ultrasound Assessment in HF Patients.

HEFESTOS Score	LUS Score (B Lines)	Clinical Interpretation	Follow-Up Recommendation
Low (≤3)	Normal/ Mild (≤15)	Low clinical risk with no signs of pulmonary congestion.	Routine follow-up. Standard periodic visits.
Low (≤3)	Moderate/ Severe (≥16)	Low clinical risk but subclinical pulmonary congestion present.	Intensify treatment. Early reassessment with LUS (within 1–2 weeks).
Moderate/ High (≥4)	Normal/ Mild (≤15)	Elevated clinical risk without evidence of congestion on LUS.	Frequent clinical reassessment. Monitor for non-congestive mechanisms.
Moderate/ High (≥4)	Moderate/ Severe (≥16)	High clinical risk with evident pulmonary congestion on LUS.	Intensive follow-up. Early treatment adjustment. Close monitoring.

## Data Availability

The dataset supporting the findings of this study is openly available. Access to this dataset is provided freely online via Google Sheets at the following link: https://docs.google.com/spreadsheets/d/1zRjVCplwiPkiiosdwmS6vbN17e7j60GjMC783P38B2U, accessed on 7 September 2025.

## Abbreviations

The following abbreviations are used in this manuscript:

HF	Heart failure
LUS	Lung Ultrasound
NYHA	New York Heart Association
ATDOM	Home care program

## References

[1] Bragazzi N.L., Zhong W., Shu J., Much A.A., Lotan D., Grupper A., Younis A., Dai H. (2021). Burden of heart failure and underlying causes in 195 countries and territories from 1990 to 2017. Eur. J. Prev. Cardiol..

[2] Savarese G., Lund L.H. (2017). Global public health burden of heart failure. Card. Fail. Rev..

[3] Díez-Villanueva P., Jiménez-Méndez C., Alfonso F. (2021). Heart failure in the elderly. J. Geriatr. Cardiol..

[4] Sicras-Mainar A., Sicras-Navarro A., Palacios B., Varela L., Delgado J.F. (2022). Epidemiología de la insuficiencia cardiaca en Espana: Estudio PATHWAYS-HF. Rev. Esp. Cardiol..

[5] Santos P.M., Freire R.B., Fernández A.E., Sobrino J.L.B., Pérez C.F., Somoza F.J.E., Miguel C.M., Vilacosta I. (2019). Mortalidad hospitalaria y reingresos por insuficiencia cardiaca en Espana. Un estudio de los episodios índice y los reingresos por causes cardiacas a los 30 días y al ano. Rev. Esp. Cardiol..

[6] Groenewegen A., Rutten F.H., Mosterd A., Hoes A.W. (2020). Epidemiology of heart failure. Eur. J. Heart Fail..

[7] Kleiner Shochat M., Fudim M., Kapustin D., Kazatsker M., Kleiner I., Weinstein J.M., Panjrath G., Rozen G., Roguin A., Meisel S.R. (2021). Early impedance-guided intervention improves long-term outcome in patientswith heart failure. J. Am. Coll. Cardiol..

[8] Sattouf A.A., Farahat R., Khatri A.A. (2022). Effectiveness of Transitional Care Interventions for Heart Failure Patients: A Systematic Review with Meta-Analysis. Cureus.

[9] McDonagh T.A., Metra M., Adamo M., Gardner R.S., Baumbach A., Bohm M., Burri H., Butler J., Čelutkienė J., Chioncel O. (2023). 2023 Focused update of the 2021 ESC guidelines for the diagnosis and treatment of acute and chronic heart failure. Eur. Heart J..

[10] Miglioranza M.H., Gargani L., Sant’Anna R.T., Rover M.M., Martins V.M., Mantovani A., Weber C., Moraes M.A., Feldman C.J., Kalil R.A.K. (2013). Lung ultrasound for the evaluation of pulmonary congestion in outpatients: A comparison with clinical assessment, natriuretic peptides, and echocardiography. JACC Cardiovasc. Imaging..

[11] Pugliese N.R., Pellicori P., Filidei F., Del Punta L., De Biase N., Balletti A., Di Fiore V., Mengozzi A., Taddei S., Gargani L. (2023). The incremental value of multi-organ assessment of congestion using ultrasound in outpatients with heart failure. Eur. Hear. J.—Cardiovasc. Imaging.

[12] Panisello-Tafalla A., Haro-Montoya M., Caballol-Angelats R., Montelongo-Sol M., Rodriguez-Carralero Y., Lucas-Noll J., Clua-Espuny J.L. (2024). Prognostic Significance of Lung Ultrasound for Heart Failure Patient Management in Primary Care: A Systematic Review. J. Clin. Med..

[13] Verdu-Rotellar J., Abellana R., Vaillant-Roussel H., Jevsek L.G., Assenova R., Lazic D.K., Torsza P., Glynn L.G., Lingner H., Demurtas J. (2021). Risk stratification in heart failure decompensation in the community: HEFESTOS score. ESC Hear. Fail..

[14] Rivas-Lasarte M., Maestro A., Fernández-Martínez J., López-López L., Solé-González E., Vives-Borrás M., Montero S., Mesado N., Pirla M.J., Mirabet S. (2020). Prevalence and prognostic impact of subclinical pulmonary congestion at discharge in patients with acute heart failure. ESC Hear. Fail..

[15] Conangla L., Domingo M., Lupón J., Wilke A., Juncà G., Tejedor X., Volpicelli G., Evangelista L., Pera G., Toran P. (2020). Lung ultrasound for heart failure diagnosis in primary care. J. Card. Fail..

[16] Domingo M., Conangla L., Lupon J., Wilke A., Juncà G., Revuelta-López E., Tejedor X., Bayes-Genis A. (2020). Lung ultrasound and biomarkers in primary care: Partners for a better management of patients with heart failure?. J. Circ. Biomarkers.

[17] Masarone D., Kittleson M.M., Pollesello P., Marini M., Iacoviello M., Oliva F., Caiazzo A., Petraio A., Pacileo G. (2022). Use of levosimendan in patients with advanced heart failure: An update. J. Clin. Med..

[18] Idescat: Institut d’Estadísitica de Catalunya (2024). Indicadors demogràfics i de territori 2024. Estructura per edats, envelliment i dependència. Anuario Estadístico de Cataluña. https://www.idescat.cat/pub/?id=inddt&n=915&geo=at.

[19] INE: Instituto Nacional de Estadística (2024). Indicadores de Estructura de la Población. Índice de Envejecimiento por Comunidad Autónoma. https://www.ine.es/jaxiT3/Datos.htm?t=1452#_tabs-tabla.

[20] Idescat: Institut d’Estadísitica de Catalunya (2022). Anuari estadístic de Catalunya. Renda familiar disponible bruta. RFDB i RFDB per habitant. Comarques i Aran, i àmbits. https://www.idescat.cat/indicadors/?id=aec&n=15917.

[21] Catalan Health Service, Generalitat de Catalunya (2021). Conceptual bases of the model of care for people with frailty and advanced complex chronic conditions. https://scientiasalut.gencat.cat/bitstream/handle/11351/7007/bases_conceptuals_model_atencio_persones_fragils_cronicitat_complexa_avancada_2020_ang.pdf.

[22] Jambrik Z., Monti S., Coppola V., Agricola E., Mottola G., Miniati M., Picano E. (2004). Usefulness of ultrasound lung comets as a nonradiologic sign of extravascular lung water. Am. J. Cardiol..

[23] Picano E., Scali M.C., Ciampi Q., Lichtenstein D. (2018). Lung ultrasound for the cardiologist. JACC Cardiovasc. Imaging.

[24] Bekgoz B., Kilicaslan I., Bildik F., Keles A., Demircan A., Hakoglu O., Coskun G., Demir H.A. (2019). BLUE protocol ultrasonography in Emergency Department patients presenting with acute dyspnea. Am. J. Emerg. Med..

[25] Wooten W.M., Hamilton L.A. (2019). Bedside ultrasound versus chest radiography for detection of pulmonary edema: A prospective cohort study. J. Ultrasound. Med..

[26] Gullett J., Donnelly J.P., Sinert R., Hosek B., Fuller D., Hill H., Feldman I., Galetto G., Auster M., Hoffmann B. (2015). Interobserver ultrasound. J. Crit. Care.

[27] Chiem A.T., Chan C.H., Ander D.S., Kobylivker A.N., Manson W.C. (2015). Comparison of expert and novice sonographers’performance in focused lung ultrasonography in dyspnea (FLUID) to diagnose patients with acute heart failure syndrome. Acad. Emerg. Med..

[28] Prosen G., Klemen P., Štrnad M., Grmec S. (2011). Combination of lung ultrasound (a comet-tail sign) and N-terminal pro-brain natriuretic peptide in differentiating acute heart failure from chronic obstructive pulmonary disease and asthma as cause of acute dyspnea in prehospital emergency setting. Crit. Care.

[29] Núnez J., de la Espriella R., Rossignol P., Voors A.A., Mullens W., Metra M., Chioncel O., Januzzi J.L., Mueller C., Richards A.M. (2022). Congestion in heart failure: A circulating biomarker-based perspective. a review from the BiomarkersWorking Group of the Heart Failure Association, European Society of Cardiology. Eur. J. Heart Fail..

[30] Núnez J., Llàcer P., García-Blas S., Bonanad C., Ventura S., Núnez J.M., Sánchez R., Fácila L., de la Espriella R., Vaquer J.M. (2020). CA125-guided diuretic treatment versus usual care in patients with acute heart failure and renal dysfunction. Am. J.Med..

[31] Núnez J., Bayés-Genís A., Revuelta-López E., Ter Maaten J.M., Minana G., Barallat J., Cserkóová A., Bodi V., Fernández-Cisnal A., Núnez E. (2020). Clinical role of CA125 in worsening heart failure: A BIOSTAT-CHF study subanalysis. JACC Heart Fail..

[32] Platz E., Jhund P.S., Girerd N., Pivetta E., McMurray J.J.V., Peacock W.F., Masip J., Martin-Sanchez F.J., Miró Ò., Price S. (2019). Expert consensus document: Reporting checklist for quantification of pulmonary congestion by lung ultrasound in heart failure. Eur. J. Heart Fail..

[33] Gargani L., Girerd N., Platz E., Pellicori P., Stankovic I., Palazzuoli A., Pivetta E., Miglioranza M.H., Soliman-Aboumarie H., Agricola E. (2023). Lung ultrasound in acute and chronic heart failure: A clinical consensus statement of the European Association of Cardiovascular Imaging (EACVI). Eur. Hear. J.—Cardiovasc. Imaging.

[34] Landolfo M., Spannella F., Giulietti F., Di Pentima C., Giordano P., Borioni E., Landi L., Di Rosa M., Galeazzi R., Sarzani R. (2024). Role of NT-proBNP and lung ultrasound in diagnosing and classifying heart failure in a hospitalized oldest-old population: A cross-sectional study. BMC Geriatr..

[35] Mitchell C., Rahko P.S., Blauwet L.A., Canaday B., Finstuen J.A., Foster M.C., Horton K., Ogunyankin K.O., Palma R.A., Velazquez E.J. (2019). Guidelines for performing a comprehensive transthoracic echocardiographic examination in adults: Recommendations from the american society of echocardiography. J. Am. Soc. Echocardiogr..

[36] Volpicelli G., Elbarbary M., Blaivas M., Lichtenstein D.A., Mathis G., Kirkpatrick A.W., Melniker L., Gargani L., Noble V.E., Via G. (2012). International Liaison Committee on Lung Ultrasound (ILC-LUS) for International Consensus Conference on Lung Ultrasound (ICC-LUS). International evidence-based recommendations for point-of-care lung ultrasound. Intensive Care Med..

